# Assessment of Risk Factors of Obesity and Diet on Breast Cancer in Ankara, Turkey

**DOI:** 10.12669/pjms.326.11346

**Published:** 2016

**Authors:** Nural Erzurum Alim, Gul Kiziltan

**Affiliations:** 1 Nural Erzurum Alim, Ph.D. Republic of Turkey Ministry of Health, Turkey Public Health Institutions, Department of Cancer, Sihhiye, Ankara, Turkey; 2 Prof. Gul Kiziltan, Department of Nutrition and Dietetics, Baskent University of Health Science Faculty, Etimesgut, Ankara, Turkey

**Keywords:** Body Mass Index, Breast cancer, Dietary habits

## Abstract

**Objective:**

To determine the risk factors of obesity and diet on breast cancer in Ankara, Turkey.

**Methods:**

A case-controlled study was carried out on newly diagnosed 40 breast cancer patients [patient group (PC)] and 40 volunteer individuals [control group (CG)] with no diagnosis of cancer and history of cancer in the family with similar characteristics to the age and gender-matched patient group between March and July 2016. All the individuals were administered a questionnaire by face-to-face interview method.

**Results:**

The mean menarche age, age at first birth and menopause age were 13.0±1.17, 22.6±3.78 and 44.33±2.39 years in PG and 12.3±0.95, 21.6±2.99, 46.71±2.41 years in CG, respectively. The mean BMI values were determined as 28.1±6.75 kg/m^2^ in PG and 30.1±6.18 kg/m^2^ in CG (p>0.05). It was determined that intake of vitamin C and fiber decreases the risk of breast cancer. Also, eating quickly and smoking were risk factors for breast cancer (p<0.05).

**Conclusion:**

This study indicated that there are relationships between menarche age, menopause age, and age at first birth, eating quickly, smoking and breast cancer. Conversely, there are significant negative relationships between dietary fiber, vitamin C intake and breast cancer. As a result, it can be said that there is a link between breast cancer and lifestyle factors and a reduction in the risk of developing breast cancer can be achieved through changes in diet, one of the lifestyle factors.

## INTRODUCTION

Breast cancer is known to be the most commonly observed cancer type in Turkey and in the world.^1^ At the same time, it is the most common reason for cancer-induced death among women.^2^ Breast cancer incidence is higher in developed countries than developing countries, whereas mortality rates are lower.^2^ The remarkable geographical differences in breast cancer incidence rates may be associated with genetic diversity across populations and differences in lifestyle including diet or environmental exposures.^3^ Besides, although it is known that lifestyle and environmental factors have a significant role in breast cancer risk, findings as to the influence of factors associated with diet on breast cancer risk is inconclusive.^4^ According to the definition by the World Health Organization, being overweight and obesity is abnormal or excessive fat accumulation that poses a risk in terms of health.^5^ Being overweight and obesity is the principal risk factor for a variety of chronic diseases, which include diabetes and cancer.^6^ Among postmenopausal obese women, estrogens with higher bioavailability are caused by increasing levels of adrenal androgen conversion to estrogens through aromatase and decreasing levels of circulating sex hormone binding globulin. It is more likely for premenopausal women who are obese to experience anovulation. There is a link between high values of body mass index (BMI) and higher levels of estradiol, estrone and testosterone in circulation in postmenopausal women who have and do not have breast cancer. However, BMI functions as a breast cancer risk factor regardless of the levels of serum estrogen, which suggests that breast cancer risk is influenced by mechanisms apart from the estrogen stimulation of the breast. It appears that women who have breast cancer and who have estrogen receptor positive (ER+) tumors have higher mortality rates due to obesity. However, this is not the case with the women with estrogen receptor negative (ER) tumors. It has also been asserted that hormones of metabolism (in the leptin family), inflammatory mediators (C-reactive protein (CRP), adiponectin, prostaglandins), depressed immune system functioning (T helper cells, leukocytes, NK cells) and reactive oxygen species (ROS) explain the reason that obese women who have breast cancer diagnosis have a more negative outcome compared to their slimmer counterparts.^7^

The influence of dietary factors on breast cancer risk have been investigated extensively. Up to now, however, the findings have been mostly inconclusive and inconsistent.^8^ The Project of European Prospective Investigation into Cancer (EPIC), involving approximately 520,000 participants across 10 European countries, is investigating the association between lifestyle and cancer. The aim of EPIC is to report about the relation of diet and a number of cancer types, including breast cancer, in the following 10-20 years. According to the findings of EPIC, the risk of breast cancer among females who consumed higher amounts of saturated fat was higher than those who consumed the lowest amount.^9^ This study was aimed to assess the risk factors of obesity and diet and their effects on breast cancer in Ankara, Turkey.

## METHODS

This case-controlled study was carried out on newly diagnosed 40 breast cancer patients aged 18 years and over who applied to Hacettepe University Faculty of Medicine Oncology Hospital Medical Oncology Unit [patient group (PC)], and 40 volunteer individuals with no diagnosis of cancer and no cancer history in the family, who had similar characteristics to the age and gender-matched patient group and who applied to Hacettepe University Department of Internal Medicine [control group (CG)] between March and July 2016. All the individuals included in the scope of the study were provided a questionnaire composed of three parts (demographic characteristics, general and dietary habits) by face-to-face interview method. The cases and controls were patients who visited hospital for the study. All the patients who came to the hospital and volunteered were taken into the sample since the number of patients involved in the study was limited. Maximum number of patients that can be reached within the permitted duration of the study was achieved. The study was approved by the Institutional Review Board and Ethics Committee of Baskent University (Project No: KA15/22) and all subjects gave written consent.

### Assessment of dietary intakes

The nutritional status of the participants was determined by food frequency questionnaire. The portion sizes of the food items were determined by means of a picture booklet consisting of 120 food images. The energy and nutrition values were assessed using the Nutrient Base program (BEBIS).^10^

### Assessment of anthropometric measurements

Body weight of the participants was measured with light clothes on, but without socks and shoes by Tanita Body Composition Analyzer UM-073. Height was measured in a standing position with head at Frankfort plane using the Seca 206 mechanical measuring tape, which is a commercial stadiometer. The following formula was used in order to calculate the Body Mass Index (BMI): weight (kg)/height (m^2^).^6^ Exclusion criteria included pregnancy, any type of cancer history and being aged below 18, which is known to influence nutritional status.

### Statistical analysis

The SPSS version 20.0 for Windows was used in order to perform the statistical analysis. The results were displayed as mean±standard deviations (±SD), frequencies and percentages. Pearson chi-square (χ^2^) test was used to compare the demographic characteristics of the participants. In the cases when there were dichotomous dependent variables, unconditional logistic regression analyses were performed in order to identify statistical differences across the groups. The relative risks were calculated as odds ratio (OR) with 95% confidence intervals (CIs). The differences that had a probability value of p<0.05 were deemed significant.

## RESULTS

The demographic characteristics of the study participants are shown in Table-I. The mean age of women in the PG and CG was similar (51.8±12.90 years and 50.9±13.05 years). The educational status of the PG is mostly high school and above compared to the CG. The majority of the participants (55.0% in the PG and 60.0% in the CG) were housewives. The 90% in the PG and 85.5% in the CG were married.

**Table-I:** Demographic characteristics of the participants.

Demographic characteristics	Patient Group (n:40)		Control Group (n:40)	

	n	%	n	%
*Age (years)*				
≤ 29	1	2.5	1	2.5
30 - 39	6	15.0	8	20.0
40 - 49	10	25.0	9	22.5
50 - 59	11	27.5	11	27.5
≥ 60	12	30.0	11	27.5
x̄± SD	51.8 ± 12.90		50.9 ± 13.05	
Min-max	23 – 83		22 – 84	
	*X*^2^ = 0.382		P = 0.984	
*Educational Status*				
Primary school	14	35.0	21	52.5
Secondary school	1	2.5	3	7.5
High school	12	30.0	4	10.0
University	13	32.5	12	30.0
*Occupation*				
Unemployed	22	55.0	24	60.0
Government official	6	15.0	3	7.5
Self-employed	1	2.5	1	2.5
Others	11	27.5	12	30.0
*Marital Status*				
Married	36	90.0	34	85.0
Single	4	10.0	6	15.0

The associations between breast cancer occurrence and reproductive factors is shown in Table-II. The menarche age, age at first birth was significantly higher, the mean menopause age was lower in PG than CG (p<0.05). The mean menarche age was 13.0 ± 1.17 years among women in the PG and 12.3 ± 0.95 years in the CG [OR: 1.835 (95% CI= 1.102 – 3.055)]. The mean age at first birth was 22.6 ± 3.78 years in the PG and 21.6 ± 2.99 years in the CG [OR: 1.195 (95% CI= 11.003 – 1.424)]. The median of the number of children was 2 in both PG and CG [OR: 2.488 (95% CI= 0.886 – 6.990)]. The menopausal age was 44.33 ± 2.39 years among women in the PG and 46.71 ± 2.41 years in the CG [OR: 1.744 (95% CI= 1.176 – 2.585)].

**Table-II:** Logistic regression of reproductive factors and breast cancer.

Reproductive Factors	Patient Group (n:40)	Control Group (n:40)	β	OR	95% CI	p
Menarche Age (years)	13.03 ± 1.17	12.35 ± 0.95	0.607	1.835	1.102 – 3.055	0.020*
Age at First Birth (years)	22.64 ± 3.78	21.63 ± 2.99	0.178	1.195	1.003 – 1.424	0.046*
Number of Children	2 (1 - 6)	2 (1 - 3)	0.911	2.488	0.886 – 6.990	0.084
Menopause Age (years)	44.33 ± 2.39	46.71 ± 2.41	0.556	1.744	1.176 – 2.585	0.006*

* p<0.05

Logistic regression analyses of dietary energy and some nutrients intakes, BMI and breast cancer are shown in Table-III. Dietary energy, saturated fatty acid, vitamin A and vitamin E intake did not show an important effect on breast cancer risk, while it was determined that the intake of vitamin C and fiber decreased the risk of breast cancer [for vitamin C, OR: 0.970 (95% CI: 0.956 - 0.983), for fiber, OR: 0.813 (95% CI: 0.738 - 0.897)]. The mean BMI was determined as 28.1 ± 6.75 kg/m^2^ in PG and 30.0 ± 6.18 kg/m^2^ in CG. There was no statistically significant effect of BMI on breast cancer (p>0.05).

**Table-III:** Logistic regression of dietary energy, nutrient intakes, BMI and breast cancer.

Energy and Nutrients	Patient Group (n:40) X̄± SD	Control Group (n:40) X̄± SD	β	OR	95% CI	p
Energy (kcal)	1810.7 ± 427.5	1800.8 ± 371.36	0.001	1.000	0.999 - 1.001	0.911
Saturated Fatty Acid (g)	37.3 ± 10.84	35.9 ± 9.32	0.014	1.014	0.970 - 1.060	0.530
Vitamin A (μgRE)	1541.2 ± 882.25	1516.4 ± 469.3	0.001	1.000	0.999 - 1.001	0.874
Vitamin E (mg)	11.6 ± 3.85	11.3 ± 2.87	0.023	1.024	0.899 - 1.168	0.717
Vitamin C (mg)	96.5 ± 36.34	150.5 ± 43.77	-0.030	0.970	0.956 - 0.983	0.001*
Fiber (g)	19.4 ± 5.24	27.2 ± 6.86	-0.020	0.813	0.738 - 0.897	0.001*
BMI (kg/m^2^)	28.1 ± 6.75	30.1 ± 6.18	0.541	1.719	0.400 - 7.388	0.466

* p <0.05

Eating, general habits and breast cancer risk are presented in Table-IV. It was shown that breast cancer risk was approximately 3.5 times higher in women who have fast eating speed than those who have slow eating speed [OR=3.562 (95% CI= 1.183 – 10.731)]. A total of 17 cases and 20 controls reported smoking status. The OR for breast cancer among ever-active smokers compared with never-active smokers was 1.762 (95% CI 1.036-2.997). Women in PG were more likely to have alcohol consumption than CG, but no significant effects were found on breast cancer (p>0.05).

**Table-IV:** Logistic regression of eating, general habits and breast cancer.

	Patient Group (n:40)	%	Control Group (n:40)	%	OR	95% CI	p
Eating rate							
Slow	8	20.0	18	45.0	-		
Medium	13	32.5	10	25.0	2.925	0.906 – 9.442	0.073
Fast	19	47.5	12	30.0	3.562	1.183 – 10.731	0.024*
Smoking status							
Never Active Smoker	23	57.5	20	50.0			
Ever Active Smoker	17	42.5	20	50.0	1.762	1.036 – 2.997	0.036*
Alcohol consumption							
No	32	77.5	36	85.0			
Yes	8	20.0	4	10.0	1.357	0.221 – 8.346	0.742

* p<0.05

## DISCUSSION

Breast cancer is a complex and multifactorial disease in which both environmental and genetic factors interact closely, and it also presents serious problems for public health.^11^ There are multiple factors that contribute to the development of breast cancer among humans. Some of these have been identified; however, there are still many which have not been identified as yet. Breast cancer risk is linked with certain hereditary, reproductive and hormonal factors.^12^ It is known that age is an established breast cancer risk factor. It is stated that most cases of breast cancer and the mortalities resulting from breast cancer are observed among females aged 50 and over.^13^ In this study, it was determined that about one third of individuals with breast cancer were over the age 60 and the mean age was 51.85±12.90 years. In a study, it was observed that there was an increase of 38% in the risk of breast cancer development among women aged from 50 to 64 between years 1970 and 1995. It was stated that this increase was supported by all the social groups; however, the greatest increase was observed among workers (45%) and academicians (26%).^14^ In another case-control study it was found that 31.8% of individuals diagnosed with breast cancer were in the age group 40-49 years.^15^

It is known that menarche age, age at first birth and menopause age are the risk factors for breast cancer.^16^ In a study conducted on 97 women with breast cancer and 97 age-matched, healthy Korean women, the menarche age in women who have been diagnosed with breast cancer and women in the control group were found to be 14.60 ± 1.79 years and 15.44 ± 1.90 years (p<0.01), respectively, and the mean age at first birth was 25.45 ± 5.26 years and 25.80 ± 2.97 years, respectively.^17^ In this study, menarche age, age at first birth and menopause age are found to be significant risk factors for breast cancer. In a study, the relationship between menarche, menopause, reproduction and breast cancer was investigated. Individual data taken from 117 epidemiological studies involving 118,964 women diagnosed with invasive breast cancer who did not receive menopausal hormone replacement therapy and 306,091 women without breast cancer were analyzed. It was seen that breast cancer risk increased by 1.050 times for each early age at menarche and by a smaller amount independently for each late age at menopause. It was observed that breast cancer risk was greater for premenopausal women at the same age than postmenopausal women.^18^

It has been hypothesized that diets with high amounts of fiber or which have a small fraction of fiber provide protection against breast cancer by means of certain mechanisms. Such mechanisms include the inhibition of intestinal reabsorption of estrogen excreted through the biliary system, reduction in the synthesis of estrogen by inhibition of human estrogen synthetase and a decrease in the glycemic index.^19^ The relationship between dietary intake of fiber and risk of breast cancer was examined in a meta-analysis involving 10 prospective studies. According to the findings of this meta-analysis, each 10g/day increase in fiber intake was associated with a decrease by 7% in breast cancer risk.^20^ It was also determined in this study that fiber intake minimizes breast cancer risk. It is also known that vitamins are important in terms of human health and diseases. Today it is known that vitamins have a significant role in prevention and treatment of cancer; however, definitive results have not been obtained about this issue so far.^21^ In a case-control study, the relationship between 17 micronutrients and breast cancer risk was investigated. The case group involved 289 women diagnosed with breast cancer, while the control group involved 422 women without an acute neoplastic disease. It was found that potassium, carotenoids, lycopene, folic acid, vitamin C, E and B6 were in an inverse relationship with breast cancer risk.^22^ In this study, it was shown that a significant negative association existed between vitamin C intake and breast cancer (p<0.05).

Cigarette is among significant risk factors in carcinogenesis. Smoke contains thousands of compounds including numerous mutagens such as polycyclic aromatic hydrocarbons (PAH) and nitrosamines.^23^ According to the National Breast Screening Study of Canada, 89,835 women aged 40-59 years were monitored for a mean 22.1 years and 6549 cases of breast cancer were detected. It was found that there is an association between the duration and intensity of smoking and cumulative exposure and breast cancer.^24^ In this study, a significant difference was found between the groups in terms of the status of smoking (p<0.05).

It is predicted in a projection of the future health and the economic burden obesity will impose in 2030 that there will be approximately 500,000 additional cancer cases will appear in the United States by 2030 if the current trends in obesity continues. Another finding of this analysis is that if the BMI of every adult was decreased by one percent, which corresponds to an approximate weight loss of 1 kg (or 2.2 lbs) for an average-height adult, the increase in the number of cancer cases would be prevented and about 100,000 new cancer cases would be avoided.^6^

The results of this study showed that the mean BMI was similar in groups and there was no statistically significant effect of BMI on breast cancer (p>0.05). This situation suggests that it depends on the fact that the educational levels of individuals with breast cancer are higher than the individuals in the control, they pay greater attention to their nutritional status, and they have just received a diagnosis.

### Limitations of the study

This study has a small sample size due to the difficulty of finding individuals recently diagnosed with breast cancer during the study. Furthermore, since it was carried out in one single hospital, no generalization might be made for the obtained results. Stronger results would be obtained by enlarging the sample size provided that more patients with recent breast cancer diagnosis could be reached.

## CONCLUSION

In this study, it was found that there is a significant positive relationship between breast cancer and menarche age, age at first birth, menopause age, eating quickly and smoking; on the other hand, a significant negative relationship between intake of dietary fiber and vitamin C and breast cancer. It could be said that the risk of breast cancer development might be decreased through changes in lifestyle and with a healthy and balanced diet. There is need for further studies to confirm the observations made in the present study.
